# Use of the GenoType^® ^MTBDRplus assay to assess drug resistance of *Mycobacterium tuberculosis *isolates from patients in rural Uganda

**DOI:** 10.1186/1472-6890-10-5

**Published:** 2010-08-06

**Authors:** Joel Bazira, Benon B Asiimwe, Moses L Joloba, Fred Bwanga, Mecky I Matee

**Affiliations:** 1 Department of Microbiology, Faculty of Medicine, Mbarara University of Science and Technology, P. O. Box 1410, Mbarara, Uganda; 2 Department of Medical Microbiology, College of Health Sciences, Makerere University, P. O. Box 7072, Kampala, Uganda; 3 Department of Microbiology and Immunology, School of Medicine, Muhimbili University of Health and Allied Sciences, P. O. Box 65001, Dar es Salaam, Tanzania

## Abstract

**Background:**

Drug resistance levels and patterns among *Mycobacterium tuberculosis *isolates from newly diagnosed and previously treated tuberculosis patients in Mbarara Uganda were investigated.

**Methods:**

We enrolled, consecutively, all newly diagnosed and previously treated smear-positive TB patients aged ≥ 18 years. Isolates were tested for drug resistance against rifampicin (RIF) and isoniazid (INH) using the Genotype^® ^MDRTBplus assay and results were compared with those obtained by the indirect proportion method on Lowenstein-Jensen media. HIV testing was performed using two rapid HIV tests.

**Results:**

A total of 125 isolates from 167 TB suspects with a mean age 33.7 years and HIV prevalence of 67.9% (55/81) were analysed. A majority (92.8%) of the participants were newly presenting while only 7.2% were retreatment cases. Resistance mutations to either RIF or INH were detected in 6.4% of the total isolates. Multidrug resistance, INH and RIF resistance was 1.6%, 3.2% and 4.8%, respectively. The *rpoβ *gene mutations seen in the sample were D516V, S531L, H526Y H526 D and D516V, while one strain had a Δ1 mutation in the wild type probes. There were three strains with *katG *(codon 315) gene mutations while only one strain showed the *inhA *promoter region gene mutation.

**Conclusion:**

The TB resistance rate in Mbarara is relatively low. The GenoType^® ^MTBDRplus assay can be used for rapid screening of MDR-TB in this setting.

## Background

Despite the availability of drugs to treat tuberculosis (TB), it remains the world's leading cause of death from a single infectious disease. The World Health Organization (WHO) estimates current rates of multidrug resistant TB (resistance to at least isoniazid and rifampicin) in new and previously treated cases globally at 2.9% and 15.3% respectively, with 57% of multidrug resistant tuberculosis (MDR-TB) cases coming from three high burden countries (China, India, and the Russian Federation) [1].

Uganda is currently ranked 16^th ^among the highest TB burdened countries in the world [2]. The prevalence of MDR-TB in new cases in this setting has previously been reported to be low at less than 2% [3]. However, there are recent reports that 12.7% of re-treatment cases attending the National Tuberculosis and Leprosy Program (NTLP) clinic in Kampala, the capital city of Uganda, had MDR-TB [4] and that in one peri-urban population of Kampala the prevalence of MDR-TB was 4.4% in new cases [5]. Although drug susceptibility testing is not routinely undertaken in Uganda it would be advantageous to know the drug susceptibility status especially of re-treatment patients to facilitate appropriate patient management.

Several competing technologies have been proposed for rapid detection of drug resistant tuberculosis. Some commercial assays are currently available including INNO-LiPA Rif.TB (Innogenetics N.V, Ghent, Belgium) and GenoType^® ^MTBDR (HAIN Lifesciences GmbH, Nehren, Germany) [6]. The new version of the latter assay (GenoType^® ^MTBDRplus), targeting the *rpoB *gene associated with the resistance to rifampicin (RIF) and both genes (*katG *and *inhA*) commonly associated with the resistance to isoniazid (INH) has been evaluated mainly on cultures and clinical specimens in various low incidence settings, demonstrating excellent specificity and good concordance with phenotypic drug susceptibility test (DST) results [7,8]. A recent study demonstrated the feasibility of this assay as a screening tool when applied in a high-volume public health laboratory in a high TB and HIV, but low drug resistance, incidence area [8].

This study aimed at determining the levels and patterns of anti-TB drug resistance to the two key drugs (rifampicin and isoniazid) in *M. tuberculosis *strains isolated from TB patients from the rural setting of Mbarara, South-Western Uganda, using the commercially available GenoType^® ^MTBDRplus assay.

## Methods

### Study setting

Sampling for this study was conducted between May 2007 and April 2008 in Mbarara, South- Western Uganda. This is the second most TB burdened area in the country, with an estimated TB/HIV co-infection rate of 65% [9].

### Study design

This was a cross sectional study in which all smear-positive newly diagnosed and retreatment TB patients aged ≥ 18 years presenting at the various TB clinics in the greater Mbarara area during the study period were enrolled. Three consecutive sputum samples (spot, early morning and spot) were taken from each patient according the Uganda National TB and Leprosy Programme (NTLP) guidelines. Samples were stored at 4°C at the recruitment clinics, in any case for not more than 48 hours, until transported in a cold box to the National TB Reference Laboratory (NTRL) in Kampala for processing and culture.

### Sample processing and culture

Specimens (2.5-10 ml) were processed by the standard N-acetyl L-cystein (NALC)-NaOH method [10] and concentrated by centrifuging at 4000 × *g *for 15 minutes. The sediment was reconstituted to 2.5 ml using a phosphate buffer (pH 6.8), to make the inoculum for the smears and cultures. Two Lowenstein-Jensen slants, one containing 0.75% glycerol and the other containing 0.6% pyruvate, were inoculated with the sediment and incubated at 37°C and examined weekly for growth. Cultures were considered negative when no colonies were seen after 8 weeks incubation.

### GenoType^® ^MTBDRplus assays

Identification of mutations in *rpoB*, *katG*, genes and the *inhA *promoter region associated with resistance to RIF and INH was performed on the mycobacterial cultures according to the manufacturer's recommendations. Briefly, heat thermolysates of cultures were obtained by heating cultures suspended in Tris-EDTA (TE) at 80°C for two hours followed by incubation in an ultrasonic bath for 5 minutes. Polymerase chain reaction (PCR) and subsequent hybridization steps were performed according to manufacturer's recommendations. Thereafter, strips were attached to the evaluation sheet, read and interpreted. For quality control we included known fully susceptible and resistant isolates in each run.

### Conventional drug susceptibility testing

The indirect proportion method on Lowenstein-Jensen media was performed by the NTLP for patient management at the following final drug concentrations: rifampicin, 40 μg/ml and isoniazid, 0.2 μg/ml. The NTLP kindly provided the results for comparison with the kit results.

### HIV testing

HIV-1 testing was performed using two rapid HIV tests, Unigold Recombinant HIV (Trinity Biotech, Wicklow, Ireland) and Determine HIV-1/2 (Abbott, Tokyo, Japan). Samples were tested first with Abbot Determine and reported only when negative. Positive samples were confirmed with Unigold, while discordant results were resolved by a third rapid test kit, HIV-1/2 Stat-Pak (ChemBio, Medford, NY). Pre and post test counselling was done for all consenting individuals.

### Ethical considerations

This study received ethical clearance from the research and ethics committee of the faculty of medicine of Mbarara University of Science and Technology, the Institutional Review Board of Mbarara University of Science and Technology and the National Council for Science and Technology. Patients were identified and managed according to Uganda NTLP guidelines [11]. Informed consent to participate in the study as well as permission to use isolates from samples provided were obtained from all participants before enrolment.

## Results

### Study population and samples

We enrolled a total of 167 sputum smear positive TB suspects presenting at the various TB clinics in the greater Mbarara during the study period. The samples were graded depending on AFB count in the specimen according to the WHO recommendations [12]. Of the 167 samples that were cultured 140 (83.8%) showed growth after eight weeks of incubation, 14 (8.4%) had no growth while 13 (7.8%) were contaminated. Of the 140 isolates, 15 (10.7%) were from patients whose socio-demographic data was not available, hence left out in the later study, leaving 125 isolates for molecular analysis. Of the 125 patients in the molecular study, majority 116 (92.8%) were newly diagnosed while only 9 (7.2%) had a previous history of TB treatment. The proportion of female patients was 40% (50/125) while that of males was 60% (75/125). The mean age of the study patients was 33.7 years. HIV results were available for only 81 patients who consented to testing, of whom 67.9% (55/81) were HIV sero-positive.

### Drug susceptibility results

Susceptibility testing using the **GenoType^® ^MTBDRplus assay **showed that a total of 6/125 (4.8%) isolates were resistant to INH, 4 (3.2%) resistant to RIF, while 2 (1.6%) of these isolates were resistant to both INH and RIF (MDR). Both MDR isolates were from HIV-infected female patients less than 39 years of age with new TB. A summary of patient demographic characteristics and associated drug susceptibility pattern is shown in Table 1. There was no statistical relationship between clinical and epidemiologic characteristics and anti-TB susceptibility in the study.

**Table 1:** Comparison of clinical and epidemiologic characteristics of the patients with isolates resistant to isoniazid and rifampicin by the GenoType^® ^MTBDRplus assay

		Drug susptibility pattern		
Characteristic	Number of patients	INH^a^	RIF^b^	MDR^c^
**Age**				
< 39 years	96	2	3	2
40-60 years	27	0	1	0
> 60 years	2	0	0	0

**Gender**				
Male	75	0	2	0
Female	50	2	2	2

**TB history**				
New	116	1	3	2
Retreatment	9	1	1	0

**HIV status**				
Positive	55	1	1	2
Negative	26	0	1	0
Unknown	44	1	2	0

**Smear result**				
1-10/10-100HPF	30	0	1	1
> 1AFB/HPF	95	2	3	1

^a^Monoresistance to isoniazid; ^b^Monoresistance to rifampicin; ^c^Resistance to both isoniazid and rifampicin.

### Mutations associated with RIF and INH resistance using the GenoType^® ^MTBDRplus assay

Mutations conferring resistance to either rifampicin or isoniazid were detected in 8/125 (6.4%) of samples analyzed. In all, six isolates were resistant to rifampicin, two of which showed mutation in *katG *gene and/or *inhA *promoter region indicating that they were INH resistant, hence MDR (Table 2). The rifampicin resistant isolates displayed three types of mutations: three isolates had a mutation at position D516V, two had S531L, while one of the isolates with D516V had a further two mutations, H526Y and H526 D in the *rpoβ *gene. Only one strain had a Δ1 mutation in the wild type probes and, according to the kit manufacturer's recommendation, was considered resistant. Four isolates showed resistance to isoniazid (Table 2). Mutations associated with isoniazid resistance were less compared to those seen in rifampicin resistance, with three strains having mutations in the *katG *(codon 315) gene only while one strain had a mutation in the *inhA *gene only (position (-15) in the *mabA*-*inhA *promoter). All *KatG *wild type positive probes also had mutant probes being positive, as was the case with the single *inhA *mutant. Only one isolate resistant to rifampicin showed a double pattern while all four isoniazid resistant strains showed double patterns (three in the *KatG *probes and the other in the *inhA *probes). Additionally, one strain showing resistance to rifampicin by the proportion method on Lowenstein-Jensen media did not display a similar genotype even on repeat assay (isolate 102, Table 2).

**Table 2:** Mutations associated with RIF and INH resistance in the eight resistant isolates.

	RIF susceptibility pattern			INH susceptibility pattern					
					*KatG *probes		*inhA *probes		
Study number	Indirect Method	*rpoβ *gene WT probes	*rpoβ *gene Mutant probes	Indirect Method	WT	Mutant	WT1	WT2	Mutant
08	R		D516V, H526Y, H526D	R	WT		wt	WT	C15T
29	R		D516V	S	WT		WT	WT	
47	R		D516V	S	WT		WT	WT	
102	R			R	wt	S315T1	WT	WT	
291	R	Δ8	S531L	R	wt	S315T1	WT	WT	
246	S			R	wt	S315T2	WT	WT	
248	R	Δ1		S	WT		WT	WT	
437	R		S531L	S	WT		WT	WT	

INH = isoniazid; RIF = rifampicin; R = drug resistant isolate; S = drug susceptible isolate; Indirect method = proportion method on Lowenstein-Jensen media.

## Discussion

In the current study we have determined the levels and patterns of anti-TB drug resistance to the two key anti-tuberculosis drugs (rifampin and isoniazid) in *M. tuberculosis *strains isolated from patients from the rural setting of Mbarara, South-Western Uganda, using a line probe technique, the commercially available GenoType^® ^MTBDRplus assay. This technique may thus prove suitable for use in a majority of diagnostic laboratories in TB endemic countries which do not have the capacity to undertake culture and drug susceptibility testing of *M. tuberculosis*.

We found resistance to isoniazid and rifampicin, to be 3.2% and 5.6% respectively, while MDR was 1.6% (2/125). Our result show differences compared with findings that were obtained in a previous National anti-tuberculosis drug resistance survey in Uganda of 1996-97 that indicated a primary resistance to isoniazid of 6.7%, that to rifampicin at 0.8%, and MDR of 0.5% [13]. More recently, a study in Peri-urban Kampala showed resistance to isoniazid of 8.1%, rifampicin resistance of 4.4% and MDR was found to be 4.4% [5]. These differences may probably be due to sampling strategy employed in each study. While the National survey randomly sampled districts in Uganda, the peri-urban study in Kampala looked at a single division known to be the second most TB burdened in the city, while the current study sampled patients from various villages of a rural district in western Uganda. Whereas it is generally known that resistance to rifampicin is a surrogate marker for MDRTB, this study observed a high rate of monoresistance to rifampicin. The low sensitivity of the assay for detection of isoniazid resistance is likely due to the fact that the assay targets only *katG315 *mutations while isoniazid resistance in *M. tuberculosis *strains could also involve mutations in other *katG *gene regions or in other loci. For example, mutations in the *inhA *promoter region occur in 15% to 35% of INH-resistant *M. tuberculosis *strains from some geographical locations. Another interesting observation was a higher rate of resistance amongst female patients (6/50; 12%) compared to males (2/75; 2.7%), although not quite reaching significance. This might be related to health seeking behavior, with prolonged delays in female patients (probably due to lack of control of financial resources at household levels) as has been observed by Oola [14] in Mukono district, another rural setting in Central Uganda.

Studies from neighbouring East African countries show varied results. In the only recorded study in Rwanda, resistance to isoniazid was found at 6.2%, that to rifampicin was 3.9% with all rifampicin resistant isolates being multidrug-resistant [15]. In northern Tanzania, on the other hand, a study of 111 isolates showed that 9.9% were resistant to isoniazid, 2.7% to rifampicin, while MDR was 2.7% [16]. Generally, the drug resistance rates in the current study are fairly within the range of those found in previous studies both in-country and around the region. However there is evidence of an increase in the MDR rate in Uganda in the last two studies compared to the first National survey albeit on a smaller sample. Although a number of patients were not tested for HIV and could be dually infected, two thirds of those tested were co-infected with both HIV and TB, and this is a common trend in sub Saharan Africa [17].

In the sample analyzed, GenoType^® ^MTBDRplus results indicated one isolate resistant to rifampicin having a double pattern (positive hybridization with mutant and wild type probes), while all four isoniazid resistant strains showed double patterns (three in the *KatG *probes and the other in the *inhA *probes). The double patterns are thought to be due to heteroresistance, i.e simultaneous presence of both drug resistant and susceptible TB bacilli in samples as has been hypothesized elsewhere [18]. It has also been reported that heteroresistance is an important factor which can affect the accuracy and reliability of drug susceptibility testing results by line probe assays and maybe a reason for double patterns on GenoType^® ^MTBDRplus membranes [7,19]. Heteroresistance is more likely to occur in high TB incidence areas and in cultures isolated from chronic patients as they have more opportunity to become infected with various populations of mycobacteria [20] as is the likely scenario in our setting.

## Conclusion

The *M. tuberculosis *resistance rate in Mbarara is relatively low. Additionally, we have demonstrated that the GenoType^® ^MTBDRplus assay can be used for rapid screening of MDR-TB in this setting. However, mutation regions of the *rpoβ *and *KatG *genes that are not captured on the strips of the line probe assay may be a possible limitation in the use of this technique.

## Competing interests

The authors declare that they have no competing interests.

## Authors' contributions

JB participated in the planning of the study, acquisition of samples and demographic data, culture and isolation of Mycobacteria and drafting of manuscript; BBA participated in data analysis and drafting of the manuscript, MLJ participated in general supervision of the study and critical revision of the manuscript, FB participated in performing the HAIN assays, MM participated in the conception of the study, general supervision of the study, critical revision of the manuscript. All authors read and approved the final version of the manuscript.

## Pre-publication history

The pre-publication history for this paper can be accessed here:

http://www.biomedcentral.com/1472-6890/10/5/prepub
